# Development and internal validation of a perioperative nomogram for predicting post-operative delirium in geriatric patients with intertrochanteric fractures undergoing internal fixation with pre-operative low-dose dexamethasone

**DOI:** 10.3389/fmed.2026.1846044

**Published:** 2026-06-12

**Authors:** Wanshun Liu, Yunxi Lu, Jun Chen, Yuanhao Lv, Qingde Wa

**Affiliations:** 1 Department of Orthopaedic Surgery, The Second Affiliated Hospital of Zunyi Medical University, Zunyi, Guizhou, China; 2 Department of Anesthesia, The Third Affiliated Hospital of Zunyi Medical University (The First People's Hospital of Zunyi), Zunyi, Guizhou, China

**Keywords:** dexamethasone, geriatric patients, intertrochanteric fracture, nomogram, post-operative delirium

## Abstract

**Objective:**

To develop and internally validate a nomogram specifically for predicting post-operative delirium (POD) in elderly patients undergoing internal fixation for intertrochanteric fractures, all of whom received a standardized pre-operative low-dose intravenous dexamethasone.

**Methods:**

We conducted a retrospective cohort study of 248 patients aged ≥60 years who underwent internal fixation for intertrochanteric fracture at the Third Affiliated Hospital of Zunyi Medical University between January 2023 and October 2025. All patients received 10 mg dexamethasone intravenously 30 min before surgery as part of the institutional protocol for post-operative nausea and vomiting (PONV) prophylaxis. Feature selection was performed using the Boruta algorithm, followed by multivariable logistic regression to identify independent predictors. Model performance was evaluated using area under the curve (AUC), calibration curves, and decision curve analysis (DCA) with 1,000 bootstrap resamples for internal validation. Model interpretability was enhanced using SHAP analysis.

**Results:**

The incidence of POD was 12.5% (31/248). Three independent predictors were identified: age (OR = 1.102, 95% CI: 1.029–1.187, *P* = 0.007), pre-operative monocyte count (OR = 7.566, 95% CI: 1.047–59.183, *P* = 0.047), and post-operative transfer to ICU (OR = 2.923, 95% CI: 1.164–7.308, *P* = 0.021). The nomogram constructed from these variables demonstrated good discriminative ability, with an AUC of 0.803 (95% CI: 0.729–0.876), accuracy of 0.843, sensitivity of 0.821, and specificity of 0.847. Calibration and decision curve analyses indicated satisfactory clinical utility.

**Conclusion:**

We developed the first perioperative nomogram incorporating three perioperative variables to predict POD in geriatric intertrochanteric fracture patients undergoing a standardized pre-operative dexamethasone regimen. This model may facilitate early identification of high-risk individuals within this particular patient subset and support targeted preventive strategies immediately following surgery.

## Highlights

- This study developed a nomogram for POD prediction in geriatric intertrochanteric fracture patients with pre-operative low-dose dexamethasone.
- Age, pre-operative monocyte count, and post-operative ICU transfer were identified as independent risk factors.
- The nomogram demonstrated good discriminative ability (AUC = 0.803).
- The tool enables targeted perioperative interventions to reduce POD and improve patient outcomes.

## Introduction

Intertrochanteric fractures constitute a major source of morbidity and mortality in the elderly, accounting for nearly half of all hip fractures globally (1, 2). Their incidence continues to rise with the aging population, posing significant societal and healthcare challenges (1, 3). Despite surgical internal fixation being the standard treatment to facilitate early mobilization, post-operative complications remain common and adversely affect recovery and prognosis (4, 5). Notably, the 1-year mortality following hip fracture surgery can be as high as 20–30%, with many survivors experiencing long-term functional decline (6, 7).

Post-operative delirium (POD) is an acute neurocognitive disorder characterized by fluctuating disturbances in attention, awareness, and cognition, typically emerging 2–5 days after surgery (8–10). Its incidence following hip fracture surgery varies widely (4.7%–74%) and is associated with prolonged hospitalization, increased healthcare costs, functional decline, and elevated mortality (11–13). Neuroinflammation is recognized as a key pathogenic mechanism underlying POD (14, 15).

In our institution, a single low-dose (10 mg) intravenous dexamethasone is routinely administered pre-operatively to intertrochanteric fracture patients, primarily for antiemetic purposes. Emerging evidence from randomized trials suggests that such a regimen may also attenuate systemic inflammation and potentially reduce the risk or severity of POD in geriatric orthopedic patients (16, 17). This introduces a unique clinical scenario: a potentially modifiable risk factor (systemic inflammation) is being routinely targeted preemptively.

Accurately predicting POD remains crucial for implementing stratified care. While several studies have developed effective nomograms for POD prediction in general hip fracture cohorts, a critical gap exists (18–23). Existing models were derived from populations where pre-operative dexamethasone was not a standardized intervention, potentially overlooking the unique predictive weight of inflammatory markers when the systemic response is pharmacologically modulated.

Therefore, the primary aim of this study was to develop and internally validate a disease-specific perioperative prediction nomogram tailored to this growing patient subset. We hypothesized that a model derived from this homogeneous cohort would provide optimized risk stratification for this specific clinical context.

## Materials and methods

### Patients

This retrospective cohort study was conducted at the Third Affiliated Hospital of Zunyi Medical University. We reviewed all patients admitted with intertrochanteric femoral fractures between January 1, 2023, and October 1, 2025. The study population size was determined by the available clinical data pool, and the number of predictors was selected to adhere to the rule of at least 10 events per variable (EPV) to ensure model stability. The inclusion criteria were: (1) age ≥ 60 years; (2) diagnosis of intertrochanteric femoral fracture combined with imaging and clinical symptoms; (3) surgical treatment only including internal fixation; (4) received intravenous 10 mg (2 ml) dexamethasone in 30 min before operating. Exclusion criteria were: (1) pre-existing delirium or documented cognitive impairment pre-operatively; (2) persistent coma or inability to cooperate with assessment; (3) incomplete medical records.

### Data collection

Data were independently extracted by two investigators. Collected variables included: (1) Demographic Variables: age, sex and smoking history. (2) Clinical data: left (fracture side), other fracture, length of pre-operative hospitalization, American Society of Anesthesiologists Physical Status Classification (ASA class), diabetes, hypertension, chronic obstructive pulmonary (COPD), chronic kidney disease (CKD), cardiac arrhythmias (CA), heart failure (HF), paralysis, liver disease, stroke, malignancy, pneumonia, type of anesthesia, duration of operation, intraoperative packed red blood cell transfusions, intraoperative fresh frozen plasma transfusions, post-operative complications (pulmonary complications, respiratory infection, pneumonia), post-operative transfer to ICU. (3) Pre-operative laboratory examination results: hemoglobin (Hb), white blood cell (WBC), blood neutrophils, blood lymphocytes, blood monocytes, blood platelet (PLT), electrolytes (K, Na), serum albumin (ALB), blood AST, blood ALT, total bilirubin, direct bilirubin, blood urea nitrogen (BUN), blood creatinine (CRE), blood glucose, coagulation profile (APTT, PT, INR). (4) Pre-operative medication: benzodiazepines, NSAIDs, and Opioids. (5) Intraoperative medication: dexmedetomidine and benzodiazepines.

### Definition of POD

POD was diagnosed by the researcher and a psychiatrist by extracting the characteristic words of delirium in the patient's medical record according to the Confusion Assessment Method (CAM) scale, combined with the actual description of the delirium or delirium symptoms in the medical record. The CAM criteria require the presence of (1) acute onset/fluctuating course AND (2) inattention, plus either (3) disorganized thinking OR (4) altered level of consciousness. Patients were classified into POD or non-POD groups based on this assessment.

### Statistical analysis

Statistical analyses were performed using R software (version 4.3.2) (R Core Team, Vienna, Austria). Continuous variables are summarized as mean ± standard deviation or median (interquartile range), based on their distribution, and compared between the POD and non-POD groups using the Student's *t*-test or Mann-Whitney U test, as appropriate. Categorical variables are presented as frequencies (percentages) and compared using the chi-square test. To construct a prediction model tailored to our cohort of patients receiving pre-operative dexamethasone, the analysis proceeded in the following steps. First, to avoid overfitting and identify the most relevant predictors from our extensive dataset, we employed the Boruta algorithm—a robust wrapper feature selection method based on random forest. This step identified all features deemed potentially important for predicting POD in our specific patient population. Second, variables confirmed as “important” by the Boruta algorithm were entered into a multivariable logistic regression model. This model was used to identify independent predictors, from which odds ratios (ORs) with 95% confidence intervals (CIs) were derived. Multicollinearity among these candidate predictors was assessed using the variance inflation factor (VIF), with a VIF ≥ 10 indicating severe collinearity. Before constructing the nomogram, the individual predictive ability of each predictor was assessed by plotting ROC curve and calculating the corresponding AUC. Based on the final set of independent predictors, a nomogram was constructed to provide a visual tool for individualized risk calculation. The model's performance was rigorously evaluated across three dimensions: (1) Discrimination, quantified by the area under the receiver operating characteristic curve (AUC); (2) Calibration, assessed visually with a calibration plot and internally validated using 1,000 bootstrap resamples to correct for overoptimism; and (3) Clinical utility, evaluated by decision curve analysis (DCA) to estimate the net benefit across a range of probability thresholds. To ensure the model is robust and to correct for potential overoptimism, internal validation was performed using 1,000 bootstrap resamples. Multicollinearity was assessed using the variance inflation factor (VIF). Finally, to enhance the interpretability of the model and understand the contribution of each predictor, we performed a SHapley Additive exPlanations (SHAP) analysis.

## Results

### Baseline characteristics

**Figure 1:**
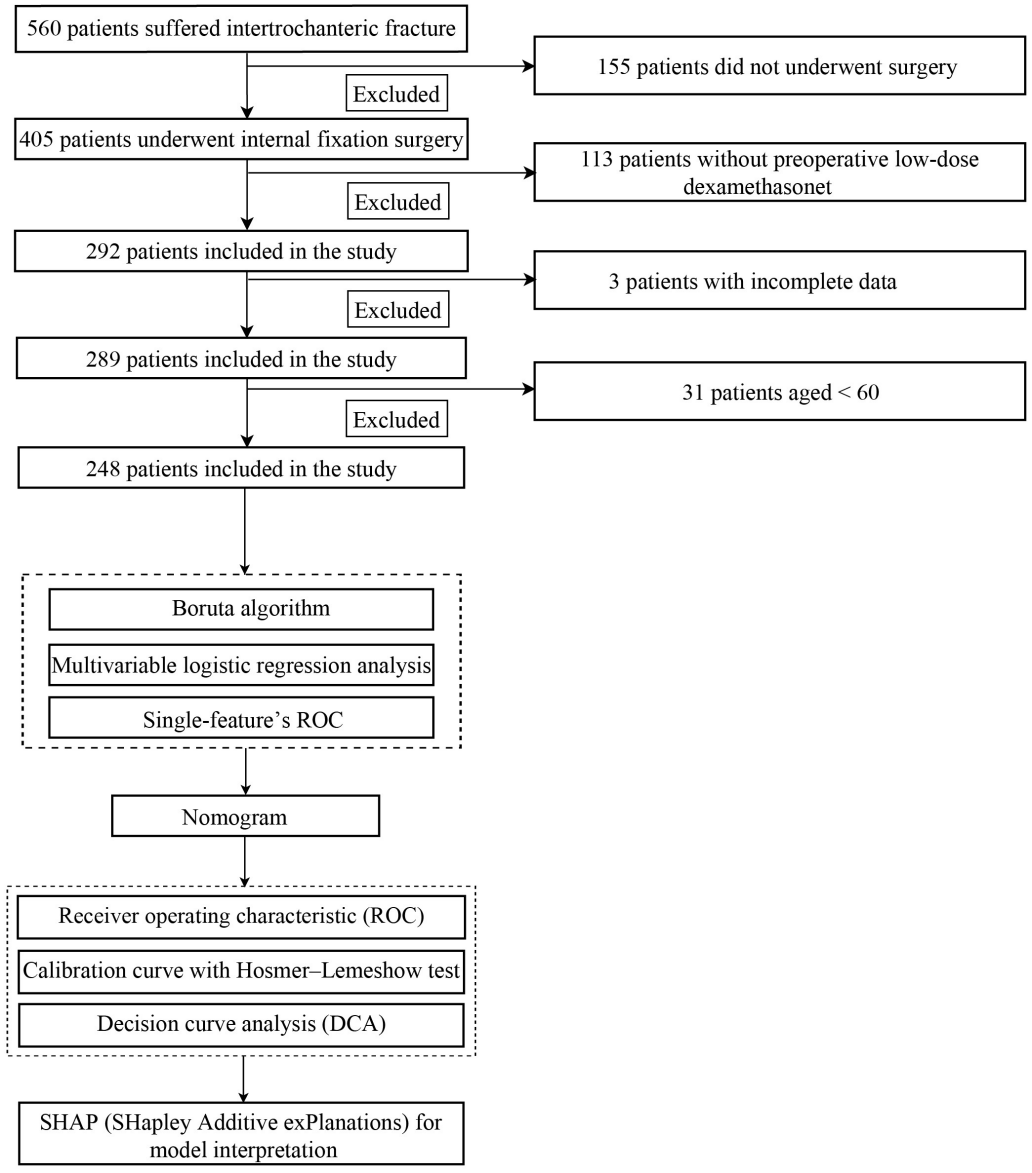
Work diagram for the study.

From an initial pool of 405 patients who underwent internal fixation, 248 eligible patients who met the inclusion criteria (including receipt of pre-operative dexamethasone) were included in the final analysis (Figure 1). The overall incidence of POD was 12.5% (31/248). As shown in Table 1, patients who developed POD were significantly older (82.7 vs. 76.4 years, *p* < 0.001), had a higher prevalence of cardiac arrhythmias (38.7% vs. 19.4%, *p* = 0.027), were more frequently transferred to the ICU post-operatively (48.4% vs. 14.7%, *p* < 0.001), and had a higher ASA classification (*p* = 0.029).

**Table 1:** Baseline clinical characteristics of the patients.

Variables	Non-delirium, *N* = 217	Delirium, *N* = 31	*P*-value
Age (year)	76.39 ± 7.68	82.71 ± 5.88	< 0.001
Gender			0.846
Female	127.0 (58.5%)	17.0 (54.8%)	
Male	90.0 (41.5%)	14.0 (45.2%)	
Smoking			0.338
No	168.0 (77.4%)	21.0 (67.7%)	
Yes	49.0 (22.6%)	10.0 (32.3%)	
Left			0.773
No	109.0 (50.2%)	17.0 (54.8%)	
Yes	108.0 (49.8%)	14.0 (45.2%)	
Other fracture			0.587
No	185.0 (85.3%)	28.0 (90.3%)	
Yes	32.0 (14.7%)	3.0 (9.7%)	
ASA			0.029
I	0.0 (0.0%)	0.0 (0.0%)	
II	41.0 (18.9%)	0.0 (0.0%)	
III	151.0 (69.6%)	26.0 (83.9%)	
IV	25.0 (11.5%)	5.0 (16.1%)	
V	0.0 (0.0%)	0.0 (0.0%)	
Diabetes			0.905
No	174.0 (80.2%)	24.0 (77.4%)	
Yes	43.0 (19.8%)	7.0 (22.6%)	
Hypertension			0.885
No	98.0 (45.2%)	13.0 (41.9%)	
Yes	119.0 (54.8%)	18.0 (58.1%)	
COPD			0.907
No	169.0 (77.9%)	25.0 (80.6%)	
Yes	48.0 (22.1%)	6.0 (19.4%)	
CKD			0.228
No	202.0 (93.1%)	31.0 (100.0%)	
Yes	15.0 (6.9%)	0.0 (0.0%)	
CA			0.027
No	175.0 (80.6%)	19.0 (61.3%)	
Yes	42.0 (19.4%)	12.0 (38.7%)	
HF			0.555
No	212.0 (97.7%)	30.0 (96.8%)	
Yes	5.0 (2.3%)	1.0 (3.2%)	
Paralysis			0.454
No	203.0 (93.5%)	28.0 (90.3%)	
Yes	14.0 (6.5%)	3.0 (9.7%)	
Liver disease			1.000
No	213.0 (98.2%)	31.0 (100.0%)	
Yes	4.0 (1.8%)	0.0 (0.0%)	
Stroke			0.474
No	164.0 (75.6%)	21.0 (67.7%)	
Yes	53.0 (24.4%)	10.0 (32.3%)	
Malignancy			1.000
No	208.0 (95.9%)	30.0 (96.8%)	
Yes	9.0 (4.1%)	1.0 (3.2%)	
Pre-operative pneumonia			0.919
No	146.0 (67.3%)	20.0 (64.5%)	
Yes	71.0 (32.7%)	11.0 (35.5%)	
15.6-8,-39243ptLength of pre-operative hospitalization (day)	4.00 (3.00–6.00)	4.00 (3.00–6.00)	0.763
Type of anesthesia			0.235
Epidural anesthesia	1.0 (0.5%)	1.0 (3.2%)	
General anesthesia	216.0 (99.5%)	30.0 (96.8%)	
15.6-8,-13243ptDuration of operation (minute)	120.00 (90.00–150.00)	110.00 (80.00–160.00)	0.749
Intraoperative packed red blood cell transfusions			0.448
No	145.0 (66.8%)	18.0 (58.1%)	
Yes	72.0 (33.2%)	13.0 (41.9%)	
Intraoperative fresh frozen plasma transfusions			0.119
No	214.0 (98.6%)	29.0 (93.5%)	
Yes	3.0 (1.4%)	2.0 (6.5%)	
Post-operative pulmonary complications			0.053
No	128.0 (59.0%)	12.0 (38.7%)	
Yes	89.0 (41.0%)	19.0 (61.3%)	
Post-operative respiratory infection			0.121
No	153.0 (70.5%)	17.0 (54.8%)	
Yes	64.0 (29.5%)	14.0 (45.2%)	
Post-operative pneumonia			0.151
No	151.0 (69.6%)	17.0 (54.8%)	
Yes	66.0 (30.4%)	14.0 (45.2%)	
Pre-operative Hb (g/L)	101.06 ± 18.97	100.97 ± 19.62	0.981
Pre-operative WBC (^*^10^9^/L)	8.00 (6.30–10.20)	8.20 (6.60–10.20)	0.497
Pre-operative neutrophils (^*^10^9^/L)	6.20 (4.50–8.00)	6.10 (4.95–8.10)	0.612
Pre-operative lymphocytes (^*^10^9^/L)	1.00 (0.70–1.30)	1.20 (0.85–1.30)	0.285
Pre-operative monocytes (^*^10^9^/L)	0.60 (0.46–0.76)	0.64 (0.50–0.81)	0.119
Pre-operative PLT (^*^10^9^/L)	171.00 (124.00–208.00)	154.00 (135.00–209.50)	0.941
Pre-operative K (mmol/L)	3.90 (3.50–4.20)	3.90 (3.55–4.10)	0.753
Pre-operative Na (mmol/L)	138.90 (136.80–140.40)	139.20 (137.65–141.40)	0.159
Pre-operative ALB g/L)	35.00 (32.70–37.70)	34.20 (30.85–36.75)	0.137
Pre-operative AST (U/L)	23.00 (19.00–29.00)	22.00 (19.50–28.00)	0.920
Pre-operative ALT (U/L)	14.00 (11.00–19.00)	12.00 (10.00–17.00)	0.242
Pre-operative total bilirubin (μmol/L)	16.90 (12.70–22.50)	17.00 (12.65–20.85)	0.989
Pre-operative direct bilirubin (μmol/L)	3.60 (2.60–5.00)	3.70 (2.85–5.40)	0.814
Pre-operative BUN (mmol/L)	6.20 (5.10–7.90)	6.70 (5.25–7.55)	0.956
Pre-operative CRE (μmol/L)	67.00 (55.60–87.00)	68.60 (59.50–95.15)	0.246
Pre-operative glucose (mmol/L)	5.90 (5.00–7.00)	6.90 (5.35–7.50)	0.072
Pre-operative APTT (s)	25.90 (24.00–27.90)	26.40 (24.60–27.70)	0.454
Pre-operative PT (s)	12.00 (11.50–12.50)	12.20 (11.60–12.40)	0.352
Pre-operative INR	1.03 (0.99–1.09)	1.05 (1.00–1.07)	0.468
Post-operative transfer to ICU			< 0.001
No	185.0 (85.3%)	16.0 (51.6%)	
Yes	32.0 (14.7%)	15.0 (48.4%)	
Pre-operative medication benzodiazepines			0.225
No	195.0 (89.9%)	25.0 (80.6%)	
Yes	22.0 (10.1%)	6.0 (19.4%)	
Pre-operative medication NSAIDs			0.920
No	141.0 (65.0%)	21.0 (67.7%)	
Yes	76.0 (35.0%)	10.0 (32.3%)	
Pre-operative medication Opioids			1.000
No	149.0 (68.7%)	21.0 (67.7%)	
Yes	68.0 (31.3%)	10.0 (32.3%)	
Intraoperative medication dexmedetomidine			0.027
No	175.0 (80.6%)	19.0 (61.3%)	
Yes	42.0 (19.4%)	12.0 (38.7%)	
Intraoperative medication benzodiazepines			0.339
No	77.0 (35.5%)	15.0 (48.4%)	
Midazolam	53.0 (24.4%)	5.0 (16.1%)	
Remimazolam	87.0 (40.1%)	11.0 (35.5%)	

### Feature selection and predictor identification

**Figure 2:**
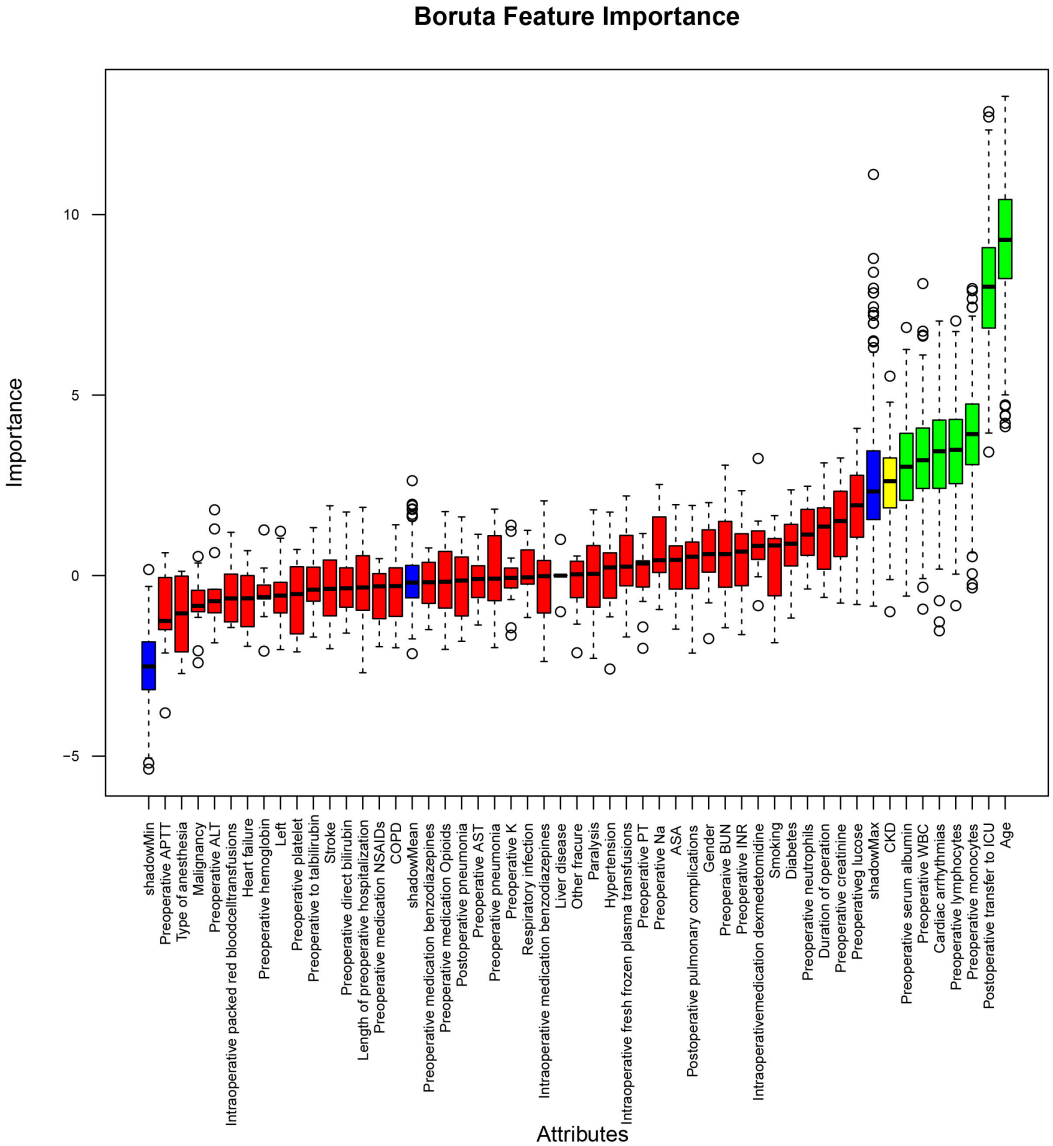
Variable selection procedure for POD prediction using Boruta's algorithm.

**Figure 3:**
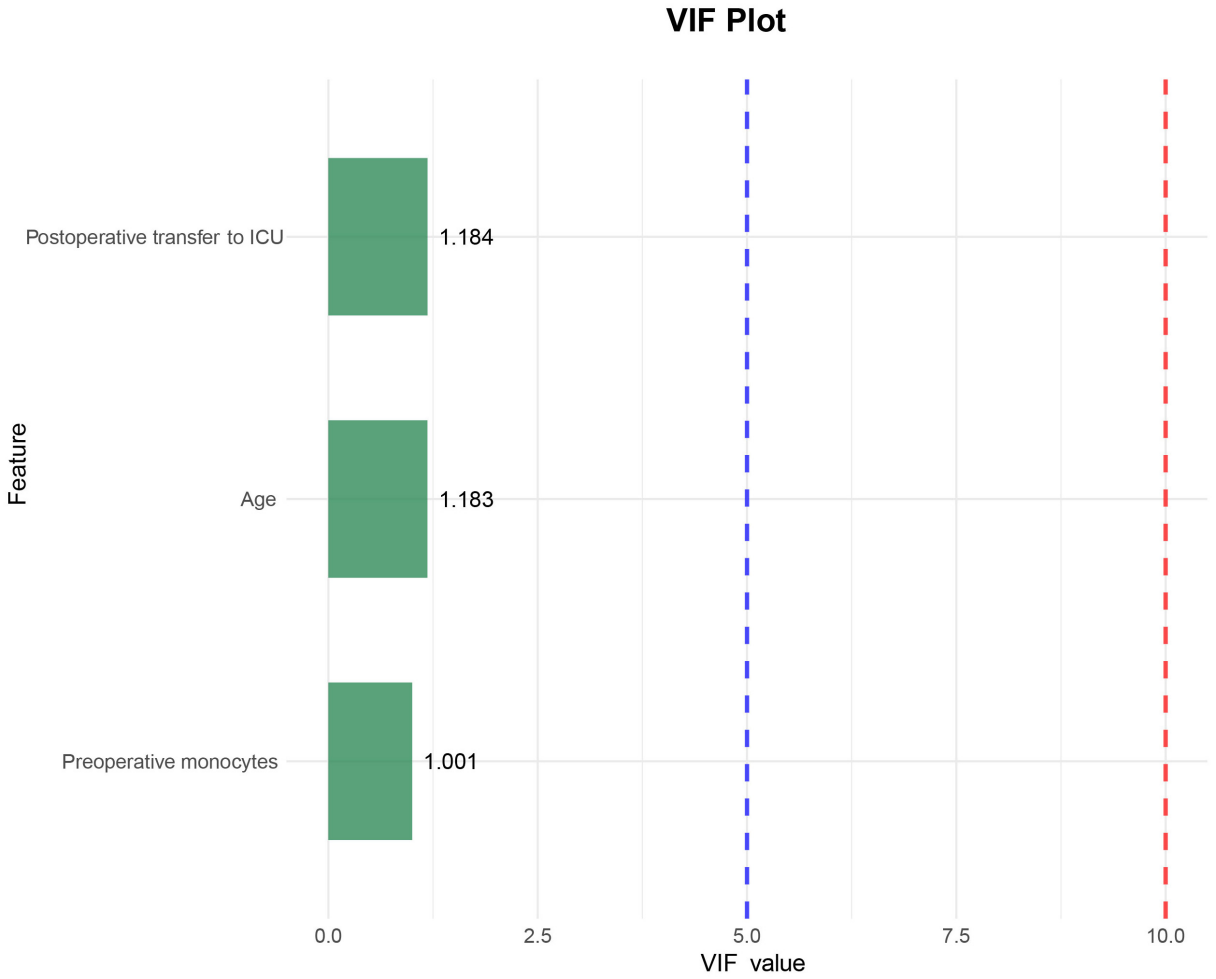
VIF plot for three variables (age, pre-operative monocytes and post-operative transfer to ICU).

**Figure 4:**
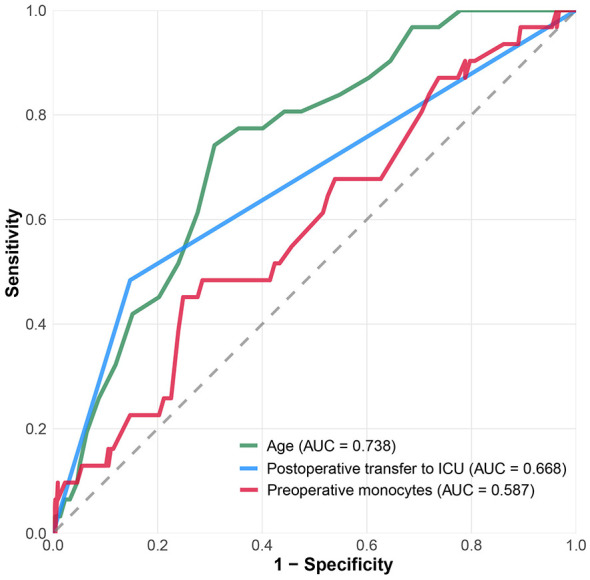
AUC values of the independently predicted by three variables (age, pre-operative monocytes and post-operative transfer to ICU).

The Boruta algorithm, after 500 iterations, identified seven variables as important: Age, Cardiac arrhythmias, Pre-operative WBC, Pre-operative lymphocytes, Pre-operative monocytes, Pre-operative serum albumin, and Post-operative transfer to ICU (Figure 2). While several variables, such as ASA class and intraoperative dexmedetomidine use, showed significant differences in baseline comparisons (*P* < 0.05, Table 1), they were not identified as important predictors by the Boruta algorithm and were thus excluded from subsequent analysis. Subsequent multivariable logistic regression analysis of these seven variables yielded three independent predictors of POD: Age (OR = 1.102, *P* = 0.007), Pre-operative monocyte count (OR = 7.566, *P* = 0.047), and Post-operative transfer to ICU (OR = 2.923, *P* = 0.021) (Table 2). No significant multicollinearity was detected among these three variables (all VIF < 10, Figure 3). The individual predictive power of each factor was limited (AUCs < 0.7, Figure 4).

**Table 2:** Multivariable logistic regression analysis of independent predictors for POD.

Variables	OR (95% CI)	*P*-value
Age	1.102 (1.029–1.187)	0.007
CA	2.048 (0.820–4.970)	0.116
Pre-operative WBC	0.974 (0.801–1.146)	0.774
Pre-operative lymphocytes	0.959 (0.350–2.364)	0.931
Pre-operative monocytes	7.566 (1.047–59.183)	0.047
Pre-operative ALB	1.014 (0.900–1.142)	0.816
Post-operative transfer to ICU	2.923 (1.164–7.308)	0.021

Variables included in the model were those retained via Boruta algorithm.

### Nomogram construction and performance

**Figure 5:**
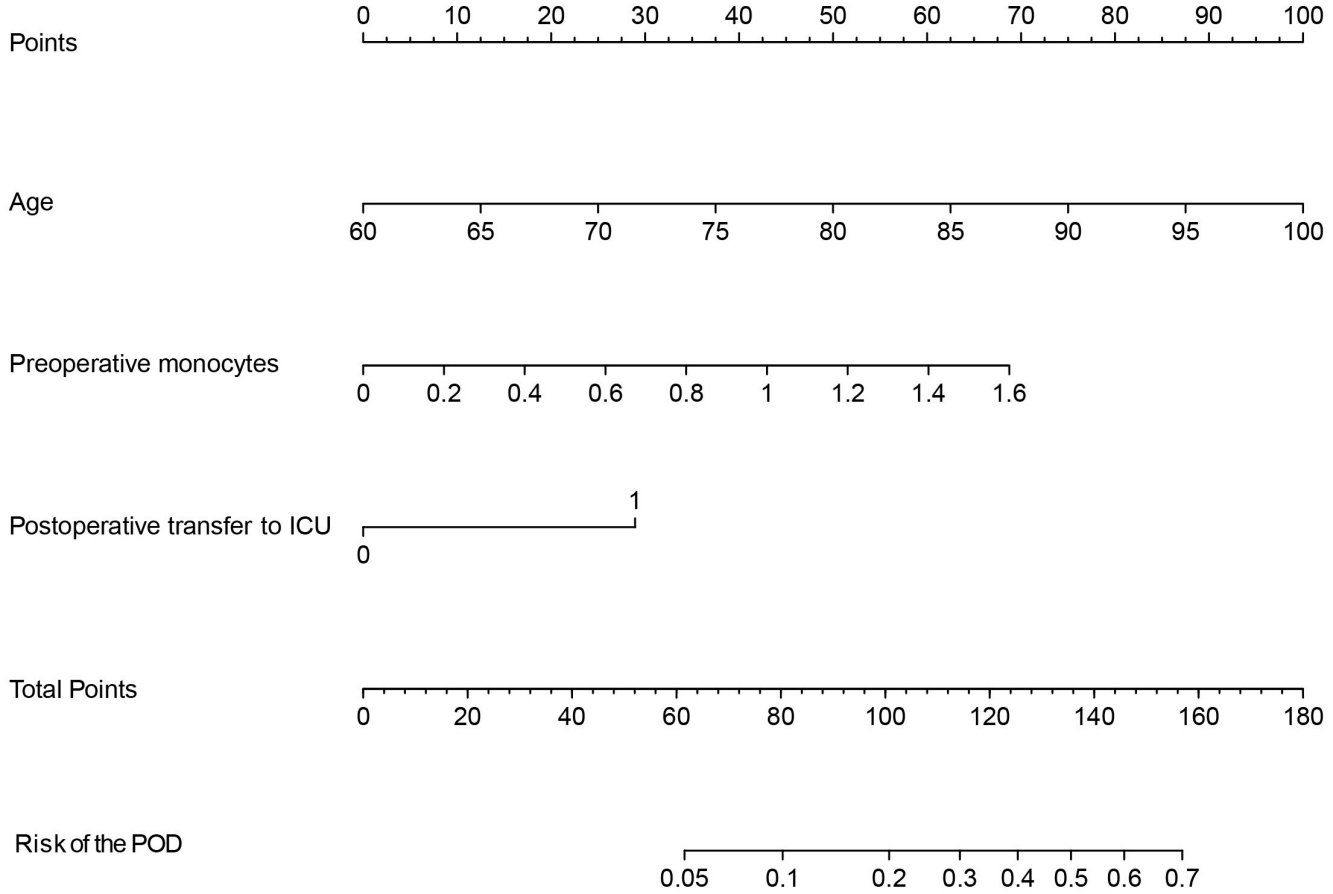
A nomogram for predicting POD based on the variables obtained from the analysis.

**Figure 6:**
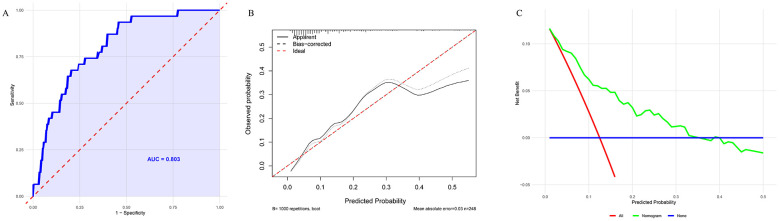
Performance evaluation of the nomogram. **(A)** Receiver operating characteristic (ROC) curve. **(B)** Calibration plot comparing predicted and observed probabilities. **(C)** Decision curve analysis (DCA) evaluating clinical utility.

A nomogram was constructed using the three independent predictors (Figure 5). The model demonstrated good discriminative ability with an AUC of 0.803 (95% CI: 0.729–0.876) (Figure 6A). The calibration curve showed good agreement between predicted and observed probabilities of POD (Figure 6B). Decision curve analysis indicated that the nomogram provided a positive net benefit across a wide range of probability thresholds, supporting its clinical utility (Figure 6C).

### Model explanation

**Figure 7:**
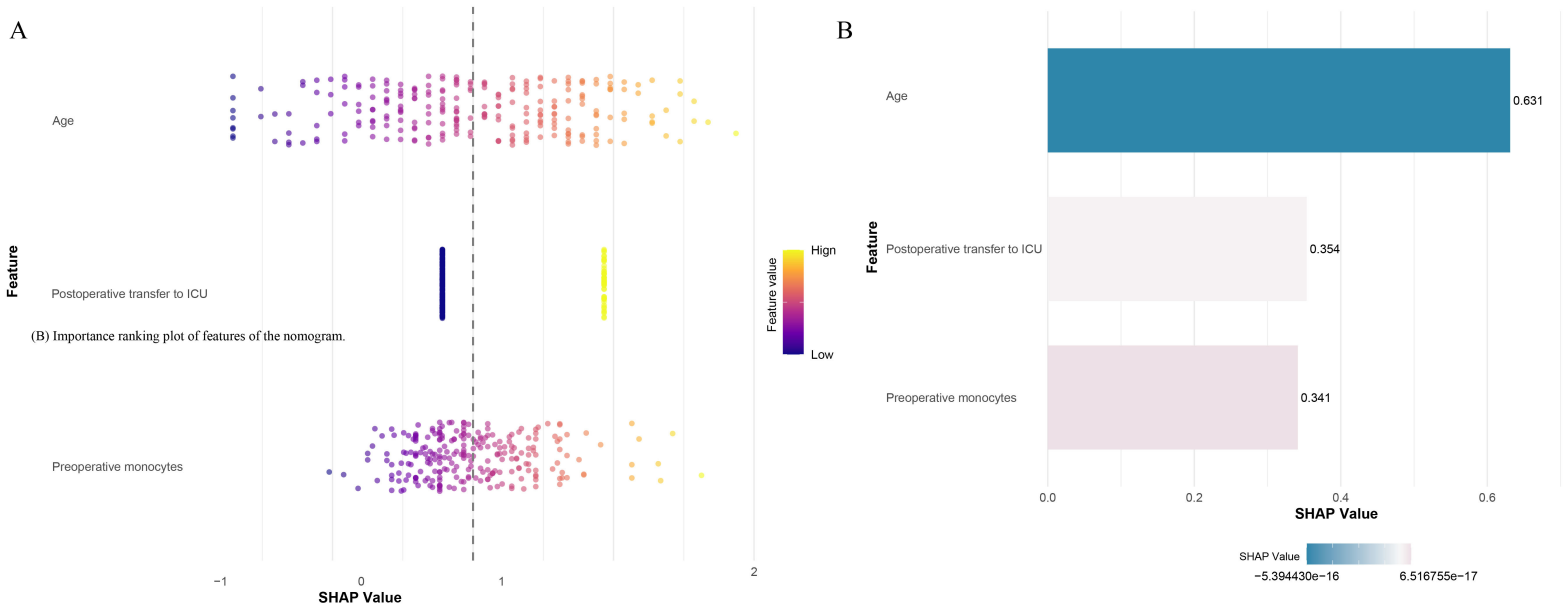
Interpretability analysis of nomogram. **(A)** SHAP dendrogram of features of the nomogram. **(B)** Importance ranking plot of features of the nomogram.

SHAP analysis was used to interpret the model. The summary plot (Figure 7) indicated that Age was the most important contributor to the model's output, followed by Post-operative transfer to ICU and Pre-operative monocyte count. Higher values of age and monocyte count, along with ICU transfer, increased the predicted risk of POD.

## Discussion

Intertrochanteric fractures in the elderly are associated with substantial functional decline, poor quality of life, and a high 1-year mortality rate despite surgical treatment (3, 24). Timely surgical fixation remains the standard of care; however, the incidence of POD after hip fracture surgery has been reported to range from 4% to 53%, making it a major perioperative complication in this population (11, 12, 25). Recent evidence suggests that pre-operative low-dose intravenous dexamethasone may help reduce the incidence and severity of POD in geriatric patients undergoing internal fixation for intertrochanteric fractures (26, 27). Against this background, early identification of high-risk patients based on perioperative clinical factors is crucial to guide targeted prophylactic strategies and optimize post-operative outcomes (22). Therefore, this study developed the first nomogram specifically designed to predict post-operative delirium in a well-defined cohort of geriatric intertrochanteric fracture patients who all received a standardized pre-operative dose of intravenous dexamethasone.

The rationale for developing a cohort-specific model stems from the potential of dexamethasone to modify the underlying risk profile for POD. Pre-operative dexamethasone is a potent anti-inflammatory agent, and neuroinflammation is a key pathway in delirium pathogenesis. Therefore, in a cohort where this inflammatory pathway is preemptively modulated, the relative importance of predictive factors may shift. Our identification of pre-operative monocyte count—a marker of innate immune activity—as an independent predictor is particularly intriguing in this context. It suggests that even under systemic anti-inflammatory coverage, a higher baseline monocytic activity may signify a residual pro-inflammatory state that predisposes patients to POD, an insight that might be obscured in more heterogeneous populations.

The discriminative performance of our model (AUC 0.803) is comparable to that of nomograms developed for broader cohorts of hip fracture patients (AUCs ranging from 0.77 to 0.85) (11, 21, 23, 28). However, the critical distinction lies not in the AUC value but in the clinical context. Previous models were designed for general risk prediction in populations without standardized pre-operative anti-inflammatory prophylaxis. In contrast, our nomogram is specifically calibrated for the growing subset of patients who have already received low-dose dexamethasone, an intervention that may fundamentally alter the inflammatory risk profile for POD. Therefore, its value is in providing context-specific risk stratification, enabling clinicians to identify high-risk individuals after a primary preventive measure has been applied, thereby guiding more nuanced secondary prevention strategies.

The predictors in our final model are clinically plausible. Advanced age is a well-established, non-modifiable risk factor. The monocyte count finding offers a potential biological link to the inflammatory hypothesis of delirium, especially relevant in our study context. Our variable selection process initially identified seven important features using the Boruta algorithm, including pre-operative WBC, lymphocytes, cardiac arrhythmias, and serum albumin. However, these factors did not retain independent significance in the final multivariable regression. Clinically, while WBC and lymphocytes are recognized markers of systemic immune status, their predictive value in our cohort was likely captured more specifically by the pre-operative monocyte count. Furthermore, factors such as cardiac arrhythmias and serum albumin are often clinical surrogates for advanced age and overall physiological frailty; in our model, their contributions were likely overshadowed by “Age” and “Post-operative transfer to ICU,” which more directly reflect baseline vulnerability and the surgical stress response. By focusing on these three independent predictors, we achieved a parsimonious model that minimizes the risk of overfitting. Regarding “post-operative ICU transfer,” while this occurs after the surgical procedure, we have defined this as a perioperative prediction tool. Given that POD typically manifests 2–5 days after surgery, identifying risk immediately upon ICU admission still provides a vital clinical window of 24–48 h. This allows clinicians to implement high-vigilance non-pharmacological interventions, such as sleep optimization and early mobilization, before symptoms emerge. Furthermore, ICU transfer often serves as a proxy for physiological frailty, surgical complexity, or early complications, all of which precipitate delirium.

This study has several limitations. First, its retrospective, single-center design may limit generalizability and introduces potential selection bias. Second, POD ascertainment relied on CAM-based keyword extraction from medical records, which is prone to underestimating the true incidence—particularly missing hypoactive delirium. Thus, our reported incidence of 12.5% likely represents a conservative estimate. Third, the sample size is modest; although it meets the requirement for stable modeling (Events Per Variable≈10), the resulting confidence interval for the AUC remains relatively wide. Finally, as all patients received dexamethasone, this study cannot assess the independent effect of dexamethasone nor directly compare the model's performance against non-dexamethasone cohorts. Given these limitations, this model should be viewed as a pilot tool, and its clinical implementation requires further multicenter external validation in similar patient populations.

## Conclusion

In conclusion, this study provides a tailored perioperative risk assessment tool for geriatric intertrochanteric fracture patients receiving standardized pre-operative dexamethasone. Future prospective, multicenter studies are essential to validate its incremental value and clinical utility through external validation.

## Data Availability

The raw data supporting the conclusions of this article will be made available by the authors, without undue reservation.
